# Mucinous Cystic Neoplasm Coexisting With a Pancreatic Pseudocyst

**DOI:** 10.7759/cureus.95947

**Published:** 2025-11-02

**Authors:** Shyamal Naidu Dharmana, Kuppusamy Senthamizhselvan, Sakthivel Harikrishnan, Roobasri Murugan, Pazhanivel Mohan

**Affiliations:** 1 Medical Gastroenterology, Jawaharlal Institute of Postgraduate Medical Education and Research, Puducherry, IND; 2 Surgical Gastroenterology, Jawaharlal Institute of Postgraduate Medical Education and Research, Puducherry, IND; 3 Pathology, Jawaharlal Institute of Postgraduate Medical Education and Research, Puducherry, IND

**Keywords:** abdominal pain, carcinoembryonic antigen, cyst fluid, mucinous cystic neoplasm, pancreatic pseudocyst

## Abstract

We report the case of a 45-year-old woman who presented with abdominal pain and distension and was initially diagnosed with a pancreatic pseudocyst. She underwent endoscopic ultrasound (EUS)-guided cystogastrostomy. Biochemical analysis of the cyst fluid revealed elevated carcinoembryonic antigen. Due to persistent symptoms and cyst fluid findings suggestive of mucinous pathology, she subsequently underwent distal pancreatectomy with splenectomy. Histopathology revealed a mucinous cystic neoplasm (MCN) coexisting within a pseudocyst. The patient had an uneventful postoperative recovery and is doing well on follow-up.

## Introduction

Pancreatic cystic lesions are heterogeneous disorders broadly classified as neoplastic and non-neoplastic [1]. Pancreatic pseudocysts are the most common cystic lesions in the pancreas, constituting 75% of cases [2]. A fibrous tissue lines them, they lack an epithelial lining, and they are non-neoplastic. Pancreatic cysts with neoplastic potential are of two types: mucinous and non-mucinous. The mucinous cysts are intraductal papillary mucinous neoplasms (IPMN) and mucinous cystic neoplasms (MCN), whereas serous cystadenomas (SCA) and solid pseudopapillary neoplasms (SPN) are non-mucinous. The mucinous cysts have the highest malignant potential. IPMN communicates with the pancreatic ductal system, whereas MCN does not [3]. MCNs may be unilocular or multilocular, and unilocular MCNs can closely mimic pancreatic pseudocysts, posing significant diagnostic challenges [4], with the management strategies differing significantly. The MCN might rarely coexist with a pseudocyst; to date, only nine cases have been reported in the literature [5]. We report a unilocular MCN coexisting with a pseudocyst, which was initially mistaken for a pseudocyst and underwent endoscopic ultrasound (EUS)-guided cystogastrostomy.

## Case presentation

**Figure 1:**
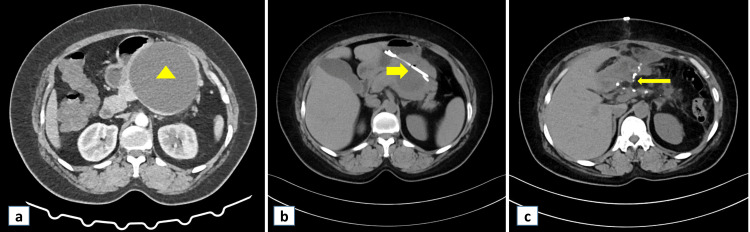
CT scan abdomen a) Arrowhead showing a single, well-defined hypodense cystic lesion of size 12.6 x 10 cm arising from the distal body and tail of the pancreas, b) the cyst size marginally decreased; broad arrow showing the cystogastrostomy stent in situ, c) follow-up CT; slender arrow pointing to no recurrence of the cyst

A 45-year-old woman presented with recurrent mid-abdominal pain and early satiety for the past three months. She noticed upper abdominal fullness for the past month. She did not have vomiting, steatorrhea, overt gastrointestinal bleed, or weight loss. There was no blunt abdominal trauma, alcohol consumption, or gallstone disease. Her past medical history was unremarkable. None of her family members had any gastrointestinal disease. On examination, she had a vague, non-tender mass in the epigastric region. There was no free fluid in the abdomen. Laboratory evaluation revealed microcytic and hypochromic anemia with hemoglobin 93 g/L (120-150 g/L), white cell count 5.9 x 10^9^ /L (4.5-11 x 10^9^ /L), platelet count 455 x 10^9^ /L (150-450 x 10^9^ /L), and serum amylase level 210 U/L (30 to 110 U/L). Contrast-enhanced computed tomography (CT) scan showed a single well-defined hypodense cystic lesion of size 12.6 x 10 cm arising from the distal body and tail of the pancreas, with a wall thickness of 2 mm (Figure 1a).

**Figure 2:**
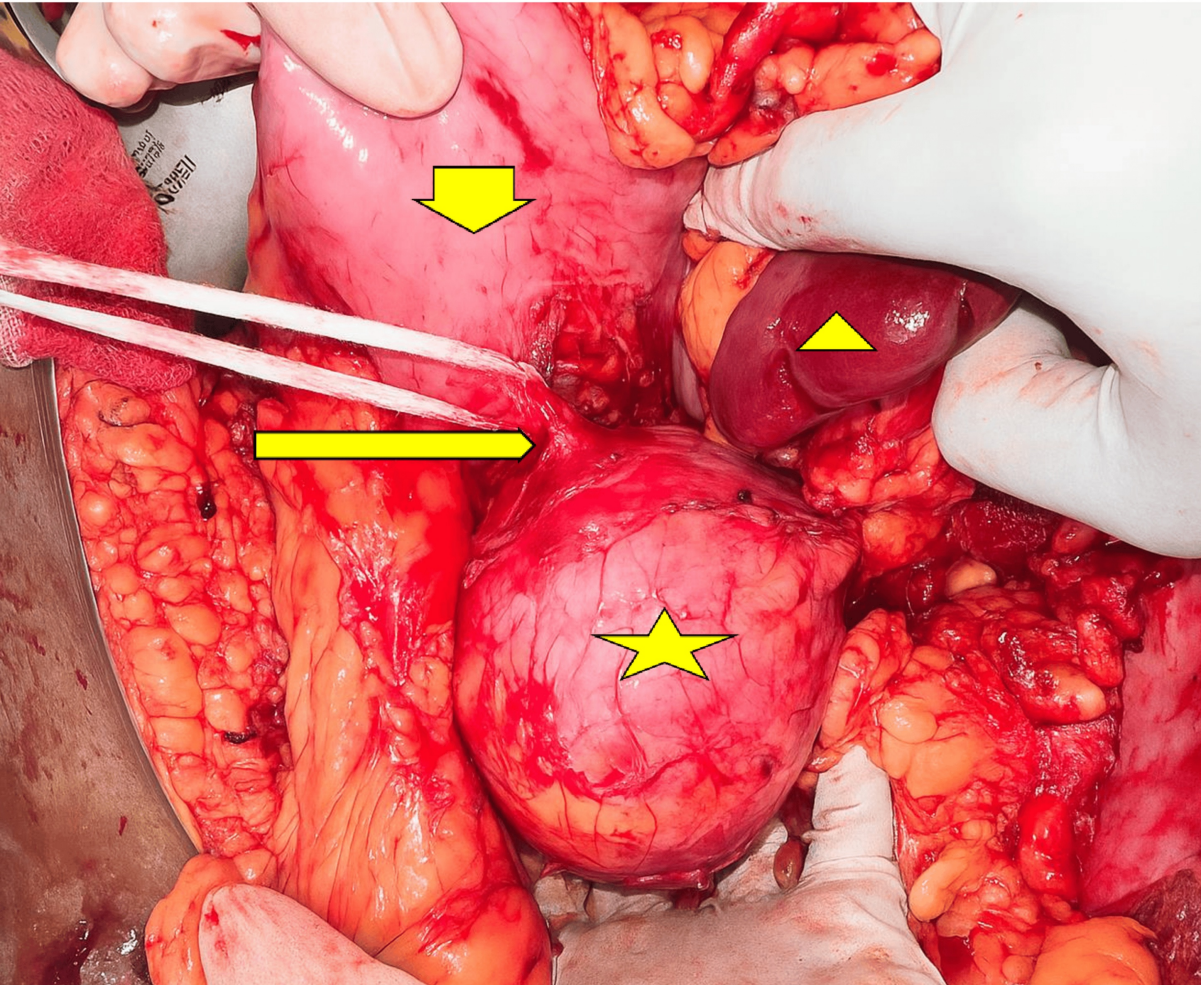
Intraoperative findings Broad arrow showing the stomach, slender arrow pointing to the cystogastrostomy site, arrowhead showing the spleen, and star showing the cyst

There was no debris inside the cyst. We considered the diagnosis of pseudocyst of the pancreas and performed EUS, which did not show septations, mural nodules, or communication with the pancreatic duct. Hence, after discussion with the family members, we proceeded with EUS-guided cysto-gastrostomy in the same sitting. We aspirated the cyst fluid during the procedure and assessed for the ‘string sign’ by stretching a drop of fluid between the gloved fingers. A positive sign (forming a long, mucinous string at least 1 cm long and lasting for at least 1 second) suggests mucinous content [6]. The ‘string sign’ was negative in our case. However, the biochemical analysis of the cyst fluid revealed a glucose value of less than 1 mg/dl, an amylase value of 14934 U/L, and carcino-embryonic antigen levels of 278 ng/ml (0-3 ng/ml). The serum CA19-9 value was 28 U/ml (0-37 U/ml). Despite cystogastrostomy, the patient continued to have abdominal pain, and a repeat CT scan showed a marginal decrease in the cyst size (Figure 1b). We discussed the case with a multi-disciplinary team and considered a diagnosis of MCN based on cyst fluid analysis. After four weeks of cystogastrostomy, the patient finally underwent distal pancreato-splenectomy with sleeve resection of the stomach (Figure 2).

**Figure 3:**
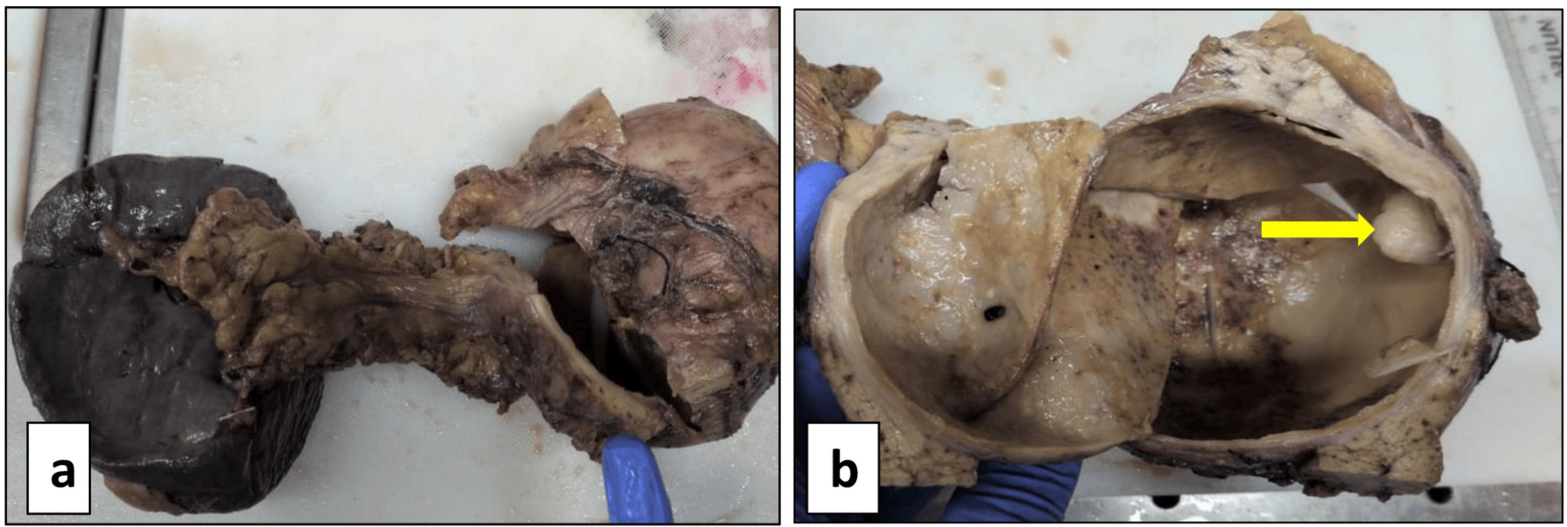
Gross specimen showing a) distal pancreato-splenectomy specimen demonstrating a well-circumscribed, thick-walled cystic lesion within the pancreas, and b) cut section of the cyst, the arrow pointing to a well-circumscribed lesion within the outer cyst wall.

The gross examination revealed a well-circumscribed, thick-walled cyst measuring 12 x 10 x 6 cm. The cut surface revealed yellowish thick material. A small cystic lesion is seen within the large cyst measuring 1 x 1 x 1 cm along the wall of the large cyst (Figure 3).

**Figure 4:**
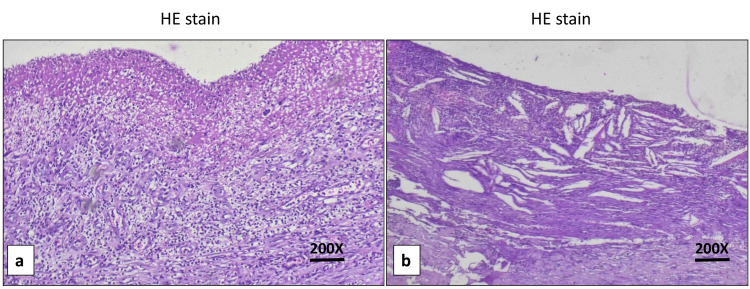
Photomicrographs showing microscopic examination of the large cyst a) The wall of a pancreatic pseudocyst lined by a fibrino-purulent exudate with acute-on-chronic inflammatory infiltrate (H and E stain, 200X), and b) the presence of cholesterol clefts associated with a mixed inflammatory infiltrate within the cyst wall (H and E stain, 200X).

Histopathological examination of a large cyst showed a fibrocollagenous cyst wall lined by a fibrino-purulent exudate with acute-on-chronic inflammatory infiltrate (Figure 4).

**Figure 5:**
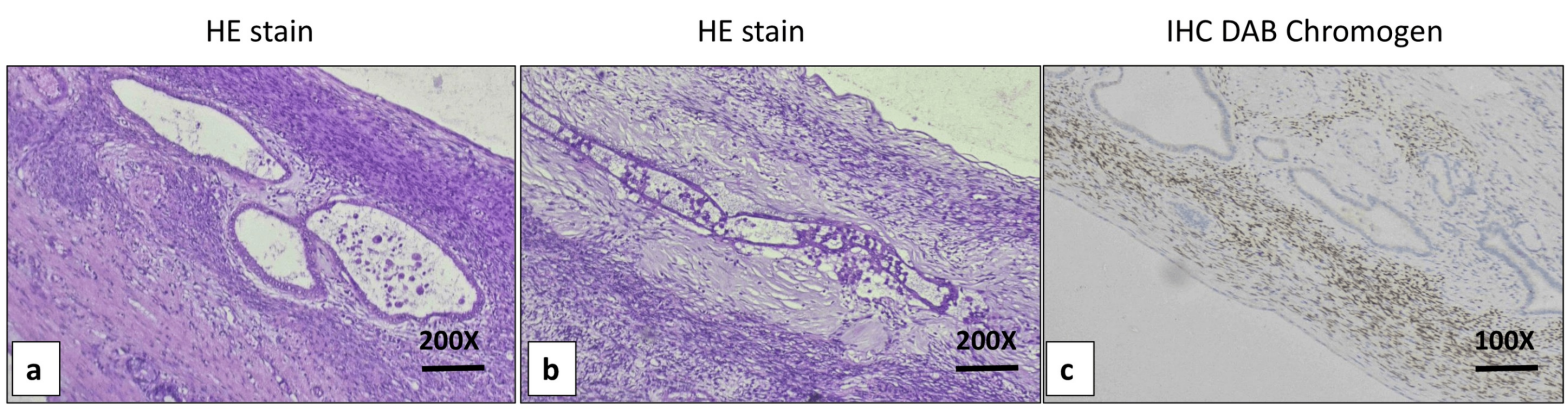
Photomicrographs showing microscopic examination of the small cyst a) Cyst wall lined by a low columnar to cuboidal epithelium with focal mucin production (H and E stain, 200X), b) the subepithelial region contains wavy spindle cells with bland nuclear features, morphologically reminiscent of ovarian stroma (H and E stain, 200X), and c) immunohistochemical staining for progesterone receptor highlights the ovarian-type stromal component (IHC, DAB chromogen, 100X).

In contrast, the small cyst was lined by low columnar to cuboidal epithelium with focal mucin. The sub-epithelium showed bland spindle cells reminiscent of ovarian stroma, which are positive for progesterone receptor on immunohistochemistry (Figure 5).

Based on these findings, we diagnosed MCN of the pancreas coexisting alongside a pseudocyst. The postoperative period was uneventful, and the patient was asymptomatic during her third month of follow-up (Figure 1c).

## Discussion

Pancreatic MCNs are rare mucin-producing cystic tumors seen among women in the fifth decade [7]. They are usually solitary, their size ranges between 5 and 35 cm, with a thick fibrotic wall, and they do not communicate with the ductal system [8]. They show a female-to-male ratio of 20 to 1 and a mean age at diagnosis between 40 and 50 years [9]. The usual location of neoplasm is in the body and tail of the pancreas in 95-98% of cases [6]. They arise from mucin-secreting epithelium supported by ovarian-type stroma and are considered premalignant lesions. Their pathogenesis is thought to involve the proliferation of mucin-producing epithelial cells and stromal activation under hormonal influence [10].

Clinically, mucinous cystic neoplasms (MCNs) are often asymptomatic and are incidentally detected during imaging for unrelated causes. When symptomatic, they most commonly present with vague upper abdominal or epigastric pain, abdominal fullness, or a palpable mass due to their large size. Some patients may experience nausea, early satiety, or back pain. Rarely, complications such as infection, rupture, hemorrhage, or pancreatitis may occur, leading to acute presentations [7].

Pseudocysts can arise following acute pancreatitis, chronic pancreatitis, or abdominal trauma. Incidence of pseudocyst in the phase of acute pancreatitis is around 5-16%, whereas it is around 20-40% following chronic pancreatitis [11]. The features of MNCs overlap with those of pseudocysts, and can be easily misdiagnosed, particularly in patients without a clear history of pancreatitis [12]. Hence, the differentiation between neoplastic cysts and pseudocysts in the pancreas remains difficult [7]. A pseudocyst in the background of a cystic neoplasm is extremely rare, and only nine cases have been reported to date (Table 1).

**Table 1:** Summary of previous reported cases of mucinous cystic neoplasms associated with a pseudocyst

Case report	Age/Sex	Presenting feature	Treatment	Follow-up
Sperti et al.,1998 [13].	Two female patients	Acute pancreatitis	Conservative treatment initially	Mucinous cystadenoma diagnosed after distal pancreatectomy.
Fischer et al.,2001 [14].	44/ Female	Acute pancreatitis/ splenic infarction	Distal pancreatectomy and splenectomy	Biopsy showed a mucinous cystadenoma.
Hsieh et al.,2002 [15].	43/ Female	Gastric outlet obstruction due to two large cysts (one was a pseudocyst and the other was a cystic neoplasm)	Distal subtotal pancreatectomy and splenectomy with en bloc resection of both lesions.	Uneventful recovery (In this case, the pseudocyst was downstream of the pancreatic duct).
Russel et al., 2005 [16].	54/ Female	Abdominal pain, recurrent pseudocyst	Exploratory laparotomy, distal pancreatectomy, and splenectomy	Biopsy showed a mucinous cystadenoma within the wall of the pseudocyst.
Yamashita et al., 2011 [17].	40/ Female	Acute pancreatitis	Observation	The cyst regressed 6 years later, and after 10 years, the cyst increased in size, and another cyst formed within the existing cyst. Underwent distal pancreatectomy.
Kamboj et al.,2017 [18].	44/ Female	Recurrent abdominal pain	EUS-guided in vivo needle-based confocal laser endomicroscopy (nCLE) suggested both a mucinous cystic neoplasm (MCN) and a pseudocyst. The patient underwent laparoscopic distal pancreatectomy and splenectomy.	Ex vivo probe-based confocal laser endomicroscopy (pCLE) and histopathology confirmed MCN.
Horiuchi et al., 2023 [5].	43/ Female	Abdominal pain	Distal pancreatectomy and splenectomy	8 years follow-up, no recurrence.
Yan et al.,2025 [19].	64/ Female	Incidental cystic lesion, tail of the pancreas	Extensive investigations suggested a pseudocyst. Conservative treatment.	Follow-up scan at 5 months showed a decrease in the size of the cyst, but 3 years later, she presented with multiple metastases.

Sperti et al. reported two cases that had been misinterpreted as a pseudocyst, and MCN was diagnosed during the follow-up [13]. Fischer et al. reported a similar case where they proposed that the small focus of precancerous epithelium may have led to obstruction of the pancreatic duct, causing formation of a pseudocyst distal to the obstruction [14]. Horiuchi et al. reported that the repeated inflammation within the MCN may have led to a large cyst formation [5]. In contrast, Hseih et al. reported a case where both pseudocyst and MCN were present simultaneously, and the pseudocyst was downstream of the pancreatic duct [15]. Russel et al. reported a concurrent presence of pseudocyst and MCN in a patient with recurrent abdominal pain [16]. There were reports of recurrence of MCN after 10 years in a patient who initially was diagnosed with acute pancreatitis with a pseudocyst, which regressed initially [17]. Kambhoj et al. used confocal laser endomicroscopy to diagnose MCN coexisting with a pseudocyst [18]. In a recent report by Yan et al., the patient presented with multiple metastases after three years of diagnosis of a cystic lesion in the tail of the pancreas, which initially regressed [19]. In our case, the small MCN probably obstructed the pancreatic duct, causing pancreatitis. If the pseudocyst had formed due to rupture of the MCN, there is a high risk of recurrence due to tumor dissemination. However, we have performed a complete resection of the cyst and sleeve gastrectomy of the cystogastrostomy site.

## Conclusions

In conclusion, MCNs can closely mimic pseudocysts, particularly when unilocular. Misdiagnosis of MCN as a pseudocyst can be prevented by integrating clinical history, detailed cross-sectional imaging, and EUS-guided cyst fluid analysis. The absence of pancreatitis, the presence of septations or a thick wall, elevated cyst fluid CEA levels, and a positive string sign should alert clinicians to the possibility of an underlying mucinous neoplasm before undertaking endoscopic drainage. The rare coexistence of an MCN and a pseudocyst underscores the importance of keeping a broad differential diagnosis for pancreatic cystic lesions, as management strategies differ significantly. A multi-disciplinary team review is prudent in doubtful cases.
